# Age- and sex-related ABC transporter expression in pyrethroid-susceptible and –resistant ***Aedes aegypti***

**DOI:** 10.1038/s41598-019-56134-2

**Published:** 2019-12-20

**Authors:** Leslie C. Rault, Ellis J. Johnson, Scott T. O’Neal, Rui Chen, Sarah E. McComic, Daniel R. Swale, Troy D. Anderson

**Affiliations:** 1 Department of Entomology, University of Nebraska, 103 Entomology Hall, Lincoln, NE 68583 USA; 2 Department of Entomology, Louisiana State University AgCenter, Baton Rouge, LA 70803 USA

**Keywords:** Metabolism, Transcription

## Abstract

Resistance mechanisms to synthetic insecticides often include point mutations and increased expression of genes encoding detoxification enzymes. Since pyrethroids are the main adulticides used against *Aedes aegypti*, which vectors pathogens such as Zika virus, understanding resistance to this insecticide class is of significant relevance. We focused on adenosine triphosphate (ATP)-binding cassette (ABC) transporters in the pyrethroid-resistant Puerto Rico (PR) strain of *Ae. aegypti*. We investigated the expression patterns of six ABC transporters previously characterized as differentially expressed in insecticide-challenged mosquitoes, or increased mRNA expression in pyrethroid-resistant *Ae. aegypti*, by comparing PR to the Rockefeller (Rock) susceptible strain. No constitutive differential expression between strains was detected, but expression differences for these genes was influenced by sex and age, suggesting that their role is independent from resistance in PR. Instead, ABC transporters may be induced after insecticide exposure. Challenging mosquitoes with deltamethrin, with or without ABC transporter modulators, showed that Rock and PR responded differently, but a contribution of ABC transporters to deltamethrin toxicity is suspected. Moreover, the effect of dexamethasone, which enhanced the inhibition of nerve firing by deltamethrin, was observed using a *Drosophila* central nervous system preparation, showing synergy of these two compounds through the potential inhibition of ABC transporters.

## Introduction

*Aedes aegypti* (L.) is a mosquito species present worldwide^1,2^ that causes important health concerns due to its ability to vector flaviviruses such as dengue, yellow fever, and Zika viruses^3,4^, as well as alphaviruses such as chikungunya virus^5^. Global efforts to control vectors of these pathogens remain inadequate or inefficient as the range of autochthonous transmission is steadily increasing globally and threatens to spread to new regions^6–9^.

Arbovirus disease mitigation predominantly relies on vector control strategies that primarily incorporate the use of synthetic insecticides, such as pyrethroids due to their enhanced safety profile. However, the prevalence of insecticide resistant mosquitoes^10–12^ has dramatically increased in disease-endemic countries and has spread to new regions of the world partly due to consistent and widespread use^13–15^. Multiple resistance mechanisms were identified in pyrethroid-resistant populations of *Ae. aegypti*, including variable point mutations in the voltage-gated sodium channel^16,17^, and increased levels of cytochrome P450 monooxygenase gene expression^18–22^. Although a growing body of literature suggests that these may be the most significant mechanisms of pyrethroid resistance, they may not be the only mechanism driving the development of pyrethroid-resistance. For instance, adenosine triphosphate (ATP)-binding cassette (ABC) transporters have been linked to transport of and/or resistance to eight synthetic insecticide classes and several mode of action groups, including carbamates, macrocyclic lactones, neonicotinoids, organophosphates, chlorinated hydrocarbons, benzoylureas, phenylpyrazoles, and pyrethroids^23,24^.

ABC transporters are a superfamily of transmembrane proteins that consist of two hydrophobic transmembrane domains (TMDs) and two nucleotide binding domains (NBDs). The ABC transporter proteins are involved in the transport of substrates across membranes using energy from ATP hydrolysis^25^. All ABC proteins function as transporters due to changes of conformation of the TMDs, which open when the NBDs hydrolyze ATP on the cytosolic side, freeing energy to allow translocation through the membrane. Moreover, the TMDs are believed to be responsible for substrate specificity^25,26^. Some ABC transporters, called permeability glycoproteins (P-glycoproteins, Pgp), have been shown to prevent anti-cancer drug accumulation and confer the phenotype of multidrug resistance (MDR) in humans and other mammals^27,28^. Another important family of ABC transporters, the ABCG transporters, have been described in different animal classes, including humans and insects. They have been shown to be involved in resistance to several anti-parasitic drugs in *Leishmania*^29,30^ and in humans due to broad substrate specificity to xenobiotic compounds, conferring drug resistance in cancer cells^31–33^.

The observations of drug export and resistance to these compounds via increased transport by ABC transporters in multiple animals suggest that their role and ability to confer resistance may be conserved across species. ABC transporters may also be a key to understanding the mode of action of currently used insecticides, and their potential role in insecticide defense in mosquitoes has been investigated for two decades. The application of verapamil, a known ABC transporter inhibitor, increased the toxicity of cypermethrin, endosulfan, and ivermectin to susceptible and organophosphate-resistant strains of *Culex pipiens* and *Culex quinquefasciatus* larvae. However, it did not increase efficacy of the organophosphate chlorpyrifos, showing that ABC transporters may not be involved in the efflux of all classes of insecticides, regardless of resistance in the strains tested^34^. Furthermore, exposure to a median lethal dose (LD_50_) of permethrin has been shown to regulate expression of ABC transporter genes on a temporal scale^35–37^ as well as between sexes in *Anopheles stephensi*^38^. These data indicate that ABC transporters play a role in the response to permethrin in mosquito and may partly contribute to the reduced insecticide sensitivity observed in resistant mosquito strains.

Porretta *et al*.^39^ examined the possible role of Pgps in defense against temephos, an organophosphate larvicide, and diflubenzuron, a growth regulator, in *Aedes caspius* larvae by applying these compounds alone or with verapamil. The LD_50_ of these insecticides was decreased when co-applied with verapamil. Similar results were observed in *Ae. aegypti* larvae exposed to temephos in presence of verapamil and confirmed by RNAi silencing of a Pgp sequence showing high similarity with sequences in *Anopheles gambiae*, *Culex quiquefasciatus*, and *Drosophila melanogaster*^40^. This observation suggests that ABC transporters are involved in the modulation of insecticide efflux, and that there may be variations between species. ABC transporters are also suspected to be contributing to insecticide resistance in *Ae. aegypti*^19,41^, although there is currently no confirmation of this hypothesis. The comparison between the pyrethroid-resistant Puerto Rico (PR) strain and the pyrethroid-susceptible Rockefeller (Rock) strain previously provided indications that the resistance mechanism involved multiple aspects, including target site mutation in the sequence of the voltage-gated sodium channel^22^ and increased P450 activity and gene expression^42^. However, the role of ABC transporters in insecticide export in *Ae. aegypti* and the previous work done in mosquitoes exposed to combinations of verapamil and insecticides also suggest that ABC transporters are significant contributors to insecticide resistance. However, verapamil is also a calcium channel blocker^43^ and could have off-target effects. Thus, another ABC transporter modulator, dexamethasone, was used. Dexamethasone has been shown to increase ABC transporter expression in rodents^44–47^. Like verapamil, dexamethasone is a substrate of ABC transporters^48,49^, which could imply that they share a similar ability to compete with other substrates of the transporters, such as insecticides, and interfere with drug efflux.

The primary goal of this study was to investigate the contribution of ABC transporters to the resistance of the PR strain of *Ae. aegypti* mosquitoes, relative to the susceptible Rock strain. A previous study suggested the upregulation of ABC transporter-encoding genes in *Ae. aegypti* mosquitoes resistant to pyrethroid insecticides^19^. However, the results of this research demonstrated no constitutive differences based on strain or exposure to deltamethrin, but did find differences based on age and sex. In toxicology experiments, treatment with ABC transporter modulators was found to alter the efficacy of deltamethrin exposure in Rock mosquitoes, and to a more limited extent in PR. Finally, a *Drosophila* central nervous system was used as a model to better understand the action of dexamethasone in combination with deltamethrin on the firing of the nerve. This model was used as an equivalent system due to the limited success of using similar preparations with the mosquito CNS. These CNS preparations also proved their reliability, as in previous studies, to test the effect of drugs on dipteran nervous systems, including *D. melanogaster* and *Musca domestica*^50–52^. Dexamethasone was found to increase the rate of nerve inhibition after exposure to deltamethrin, which suggests ABC transporters are relevant to the delivery of the insecticide to the mosquito central nervous system.

## Results

### Gene expression

#### Constitutive differences observed based on sex and age, but not strain

**Figure 1:**
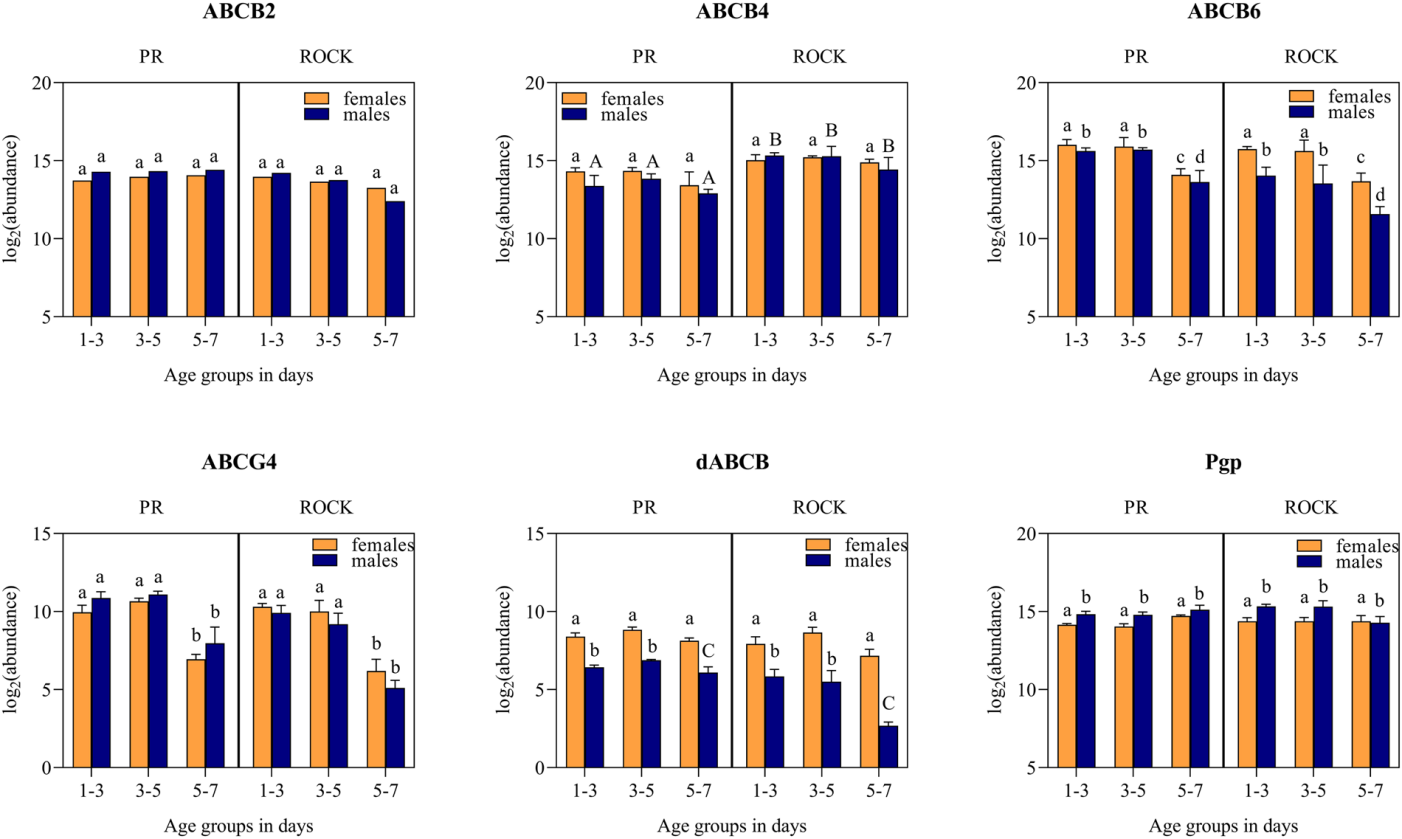
Graphical representation of the gene expression patterns, in log_2_(abundance) ± standard deviation, for the six ABC transporters of interest, comparing females and males of pyrethroid-susceptible (Rockefeller) and -resistant (Puerto Rico) *Aedes aegypti*, at all age groups. Significant differences are represented by different letters and significant interactions (Strain × Sex, and Age × Sex) are represented by upper case letters.

The gene expression analysis of six ABC transporter genes that were previously investigated in mosquitoes of various species revealed no significant differences between susceptible and resistant strains. However, significant differences in gene expression due to age and sex were observed (Fig. 1, Supplementary Table 1). More specifically, expression of the ABCG4 and ABCB6 genes showed a similar pattern in both sexes of PR and Rock, but their expression differed between the oldest age group, 5 to 7 d old, and the younger mosquitoes (Fig. 1). The expression of dABCB showed a clear difference between sexes in all age groups (Fig. 1), but the statistical analysis also highlighted a significant interaction of age, 5 to 7 d old, and sex, which can be visualized by the decrease in expression at that age in males compared to females. There were no significant differences between age groups, sexes, or strains for the gene ABCB2. The only factor with a significant effect on the expression of Pgp was sex, with a generally lower expression in females than males. The interaction of strain and sex was significant for ABCB4, which is illustrative of the larger difference in expression between sexes in PR, as females have higher expression for all age groups than Rock, where there is no difference between sexes.

#### Deltamethrin exposure did not alter ABC transporter expression

**Figure 2:**
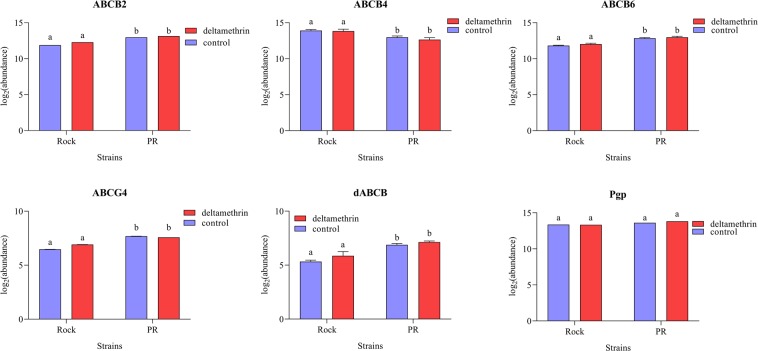
Graphical representation of the gene expression patterns, in log_2_(abundance) ± standard deviation, for the six ABC transporters of interest, comparing pyrethroid-susceptible (Rockefeller) and -resistant (Puerto Rico) *Aedes aegypti*, and exposure to deltamethrin versus an acetone control. Significant differences are represented by different letters.

The expression of all genes, except Pgp (*P* = 0.712), was significantly different between the strains (Fig. 2, Supplementary Table 2). Four of the ABC transporter transcripts, ABCB2, ABCB6, ABCG4 and dABCB, were more highly expressed in PR compared to Rock, while ABCB4 was more highly expressed in Rock with a 2.6- fold difference compared to PR. Deltamethrin treatment did not have a significant effect on the expression of any of the ABC transporter transcripts (*P*-values between 0.371 and 0.961), nor did the interaction between strain and treatment (*P*-values between 0.515 and 0.955).

### Toxicity bioassays

#### Dexamethasone and piperonyl butoxide (PBO) increased deltamethrin-induced mortality in Rock and PR, verapamil only in Rock

**Figure 3:**
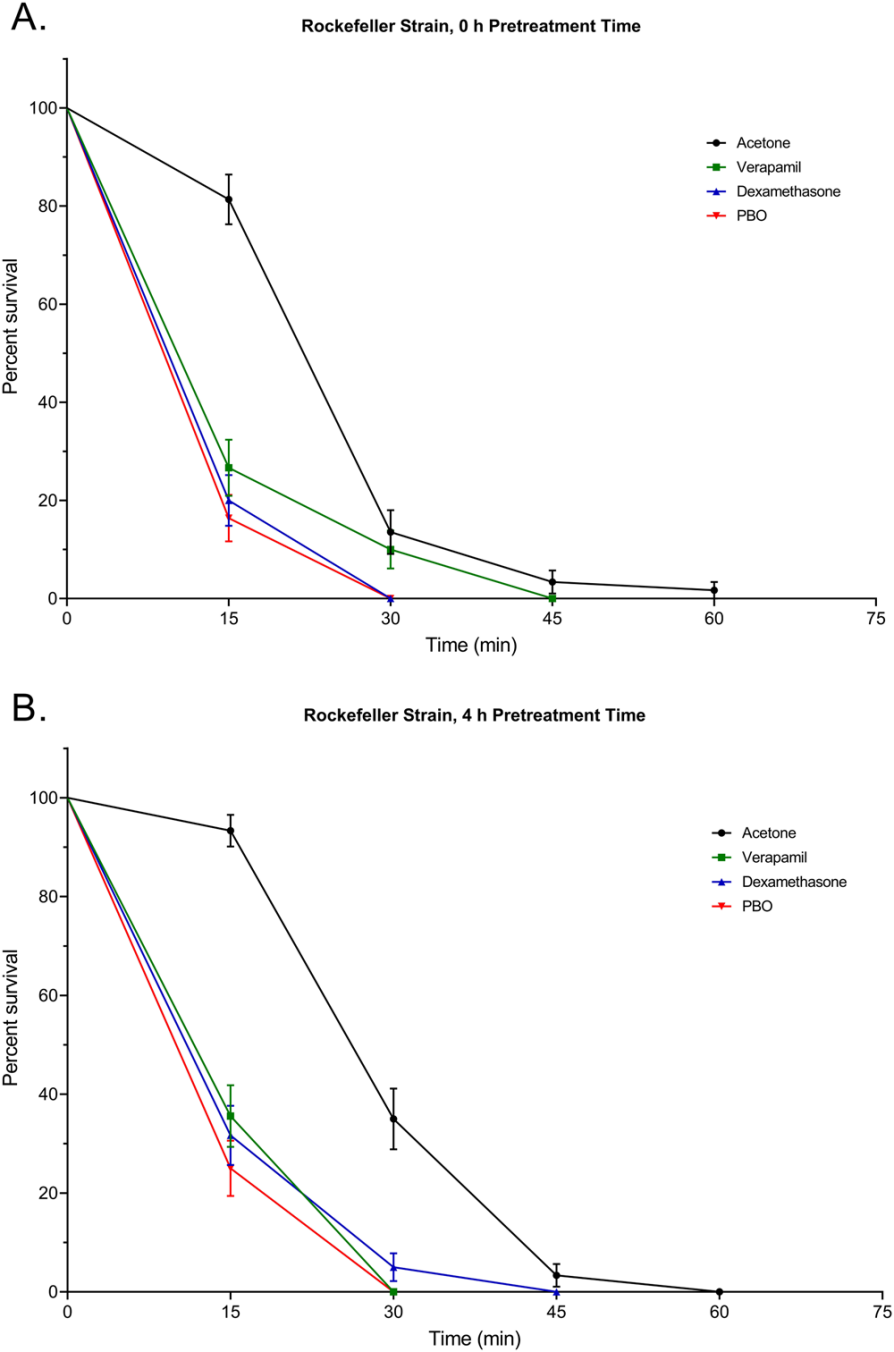
Survival of 5 to 7 d old adult, female pyrethroid-susceptible (Rockefeller) *Aedes aegypti* treated with verapamil, dexamethasone, or piperonyl butoxide (PBO) for (**A)** 0 h, or (**B)** 4 h prior to introduction into deltamethrin-coated bottles. Data presented as Kaplan-Meier survival curves with points representing mean values ± standard error for 60 mosquitoes (6 replicate groups of 10 mosquitoes).

**Figure 4:**
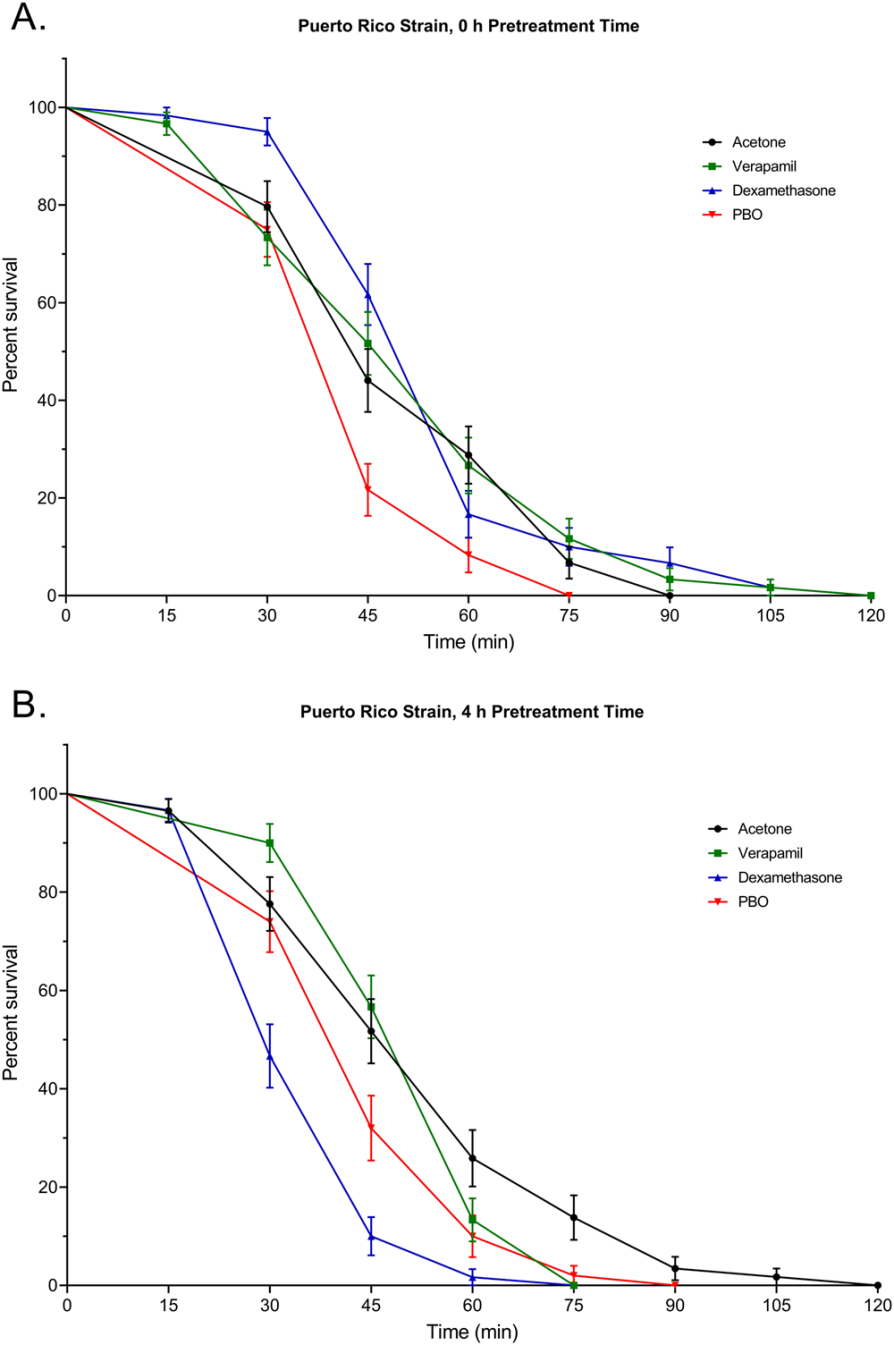
Survival of 5 to 7 d old adult female Puerto Rico mosquitoes treated with verapamil, dexamethasone, or piperonyl butoxide (PBO) for (**A)** 0 h, or (**B)** 4 h prior to introduction into deltamethrin-coated bottles. Data presented as Kaplan-Meier survival curves with points representing mean values ± standard error for 60 mosquitoes (6 replicate groups of 10 mosquitoes).

To compare the effect of verapamil and dexamethasone (Pgp-inhibitors) pretreatments followed by deltamethrin exposure on survival of the two mosquito strains, we analyzed the overall differences of all pretreatments relative to an acetone control, as well as for each treatment separately compared to the control. We used a PBO pretreatment as positive control due to its synergistic effect with deltamethrin^42^. Rock showed a significant decrease in survival (0 h pretreatment time: χ^2^ = 62.35, df = 3, *P* < 0.0001; 4 h pretreatment time: χ^2^ = 87.58, df = 3, *P* < 0.0001) compared to the acetone pretreatment control for all treatments regardless of pretreatment time (verapamil, 0 h pretreatment time: χ^2^ = 21.41, df = 1, *P* < 0.0001, 4 h pretreatment time: χ^2^ = 52.88, df = 1, *P* < 0.0001; dexamethasone, 0 h pretreatment time: χ^2^ = 45.68, df = 1, *P* < 0.0001, 4 h pretreatment time: χ^2^ = 45.70, df = 1, *P* < 0.0001; PBO, 0 h pretreatment time: χ^2^ = 50.88, df = 1, *P* < 0.0001, 4 h pretreatment time: χ^2^ = 63.40, df = 1, *P* < 0.0001) (Fig. 3). Furthermore, only PBO pretreatment in PR mosquitoes significantly differed from acetone pretreatment by decreasing survival compared to the control (χ^2^ = 8.348, df = 1, *P* = 0.0039) (Fig. 4). Pretreatments with verapamil and dexamethasone did not significantly differ from the acetone pretreatment (verapamil: χ^2^ = 0.1895, df = 1, *P* = 0.6633, dexamethasone: χ^2^ = 1.618, df = 1, *P* = 0.2033). A 4 h pretreatment time with PBO significantly decreased survival of PR relative to acetone pretreatment (χ^2^ = 5.708, df = 1, *P* = 0.0105), as did 4 h pretreatment with dexamethasone (χ^2^ = 26.99, df = 1, *P* < 0.0001), while survival after a 4 h pretreatment with verapamil did not differ from the survival after pretreatment to acetone (χ^2^ = 1.137, df = 1, *P* = 0.2862).

### Exposure to *Drosophila melanogaster* central nervous system (CNS)

#### Potency of deltamethrin and dexamethasone to firing rate of Drosophila CNS

**Figure 5:**
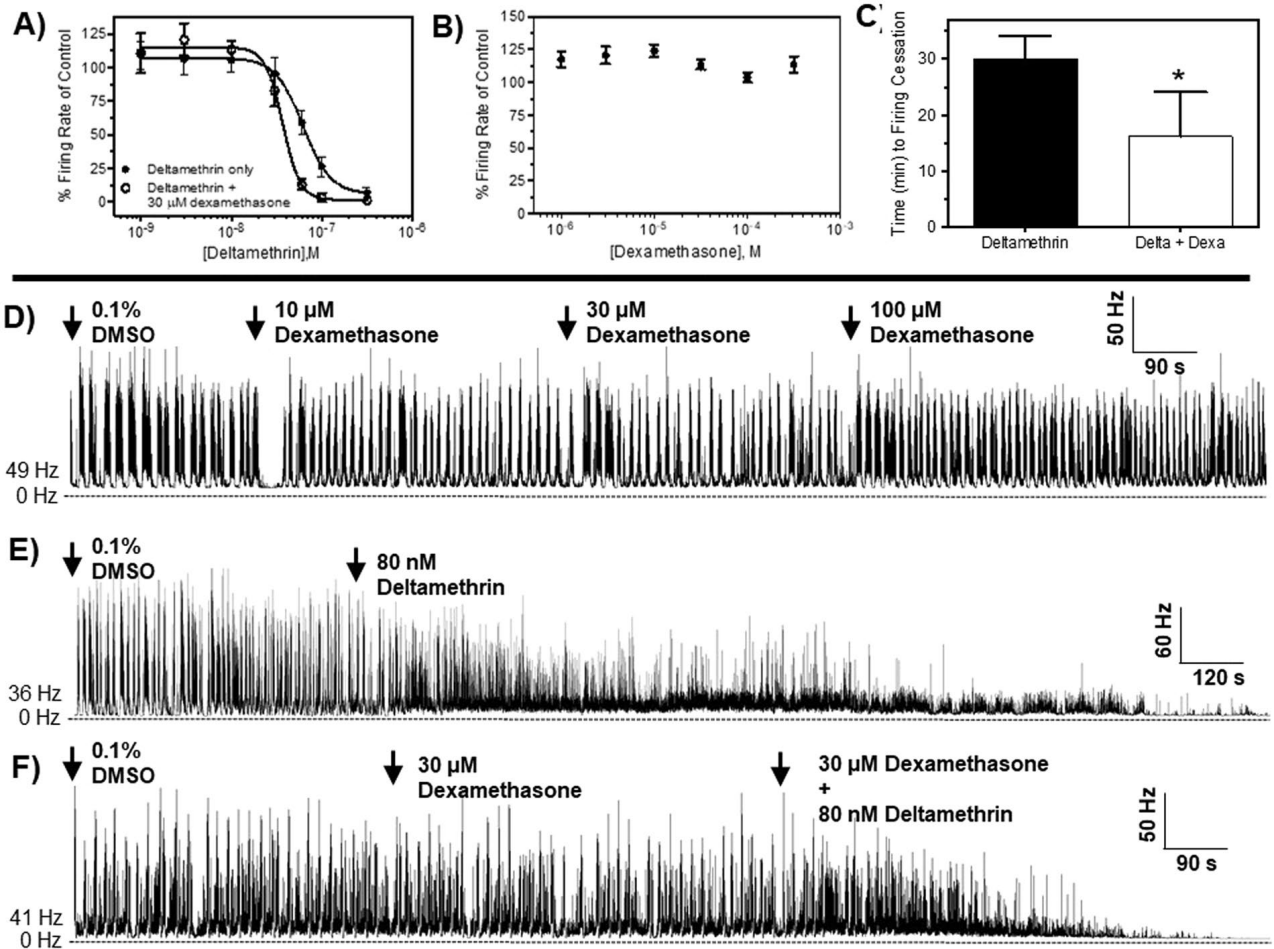
Neurophysiological recordings from the CNS of third-instar larvae of *Drosophila melanogaster*. (**A)** Effect of dexamethasone to firing rates of excised *D. melanogaster* CNS from replicated recordings (*n* = 5–8 preparations). (**B)** Concentration-response curves for deltamethrin alone (closed circles) and deltamethrin plus dexamethasone (open circles) on CNS firing rates *D. melanogaster* from replicated recordings (*n* = 5–8 preparations per curve, with each concentration replicated a minimum of 4 times). Data points in panels A-B represent mean percentage increase of baseline firing rate and error bars represent SEM. (**C)** Average (*n* = 5–10) time to cessation of CNS firing after exposure to deltamethrin and a combination of deltamethrin + dexamethasone. Asterisk represents statistical significance at *P* < 0.05 as determined by an unpaired students *t*-test. (**D–F)** Representative CNS firing frequencies before and after exposure to dexamethasone, deltamethrin, and dexamethasone + deltamethrin, respectfully. Initial firing frequencies in spikes/second (Hz) for each experiment are given to the left of each representative trace.

Deltamethrin was found to be highly potent to *Drosophila* CNS with a concentration required to inhibit 50% of firing (IC_50_) of 72 nM (95% CI: 46–81; Hillslope: −1.8; r^2^: 0.81), which is shown in Fig. 5A. Importantly, dexamethasone was found to have negligible influence on the spike discharge frequency with a maximal concentration of 300 µM increasing firing rate by 13% ± 7% (Fig. 5B,D). Based on the toxicology data, we operated around the premise that inhibition of Pgps by dexamethasone will increase the potency of deltamethrin. To test this, we pretreated an intact CNS with 30 µM dexamethasone followed by subsequent co-exposure to a range of deltamethrin concentrations. The inclusion of dexamethasone increased deltamethrin potency by 2.2-fold (IC_50_: 32 nM; 95% CI: 25–38; Hillslope: −4.1; r^2^: 0.94), which is shown in Fig. 5A. In addition to increased potency, we observed a 2.1-fold increase in Hillslope value with co-exposure of deltamethrin and dexamethasone (Fig. 5A).

#### Dexamethasone increases rate of inhibition to CNS firing after deltamethrin exposure

In addition to increased potency, we speculated that the inhibition of Pgps would reduce deltamethrin efflux from the CNS and, thus, increase the bioavailability of deltamethrin in the CNS. To test this, we measured the rate of inhibition of CNS firing after exposure to 80 nM deltamethrin, which is the approximate IC_50_ value. Data show complete inhibition of CNS firing after exposure to 80 nM deltamethrin at 30 ± 5 min post-exposure. Importantly, the rate of nerve inhibition was increased by 2-fold after co-exposure to 30 µM dexamethasone and 80 nM deltamethrin, which is a statistically significant (*P* < 0.01) increase (Fig. 5C). Representative traces showing the rate of nerve inhibition for deltamethrin alone and after co-exposure to deltamethrin and dexamethasone are provided in Fig. 5E,F.

## Discussion

This study primarily focused on determining if ABC transporters could be involved in pyrethroid resistance in PR mosquitoes by comparing various parameters to a susceptible counterpart, including constitutive gene expression, and expression after deltamethrin exposure. Additionally, the study aimed to determine if ABC transporters are involved in the response to deltamethrin exposure via toxicity in *Ae. aegypti* mosquitoes and electrophysiology bioassays via ABC transporter pharmacology with a prepared *Drosophila* CNS.

ABC transporters of the families ABCB and ABCG were considered good candidates for the export of pyrethroids out of neural tissue because they were differentially expressed in mosquitoes in response to insecticide exposure^35,36,38,39^ or as potential resistance genes^19^. In particular, ABCB transporters are known to be specific for hydrophobic substrates^53^, such as the pyrethroid deltamethrin.

Overall, there were no differences in expression of the ABC transporter transcripts of interest between PR and Rock in the experiment comparing sexes, ages and strains, but there were significant expression differences between males and females, and between age groups. Age dependent regulation of ABC transporter genes, as well as an involvement in embryo development, were previously shown in rats^54^. Sex was also a predominant factor of expression level variability, with a higher expression in female rats compared to males, resulting in quicker drug efflux by hepatic Pgps in females^55^. It was also observed that sex influenced the expression of the homologous genes in *An. stephensi*^38^.

A follow-up exposure using 5 to 7 d old female mosquitoes showed that all the ABC transporter transcripts investigated, except the transcript Pgp, exhibited significantly different expression levels between strains (Fig. 2 and Supplementary Table 2). A particularly interesting result was the expression of ABCB4, which was lower in PR mosquitoes than in Rock, contradicting previously published results^19^. This experiment also illustrates the importance of accounting for the appropriate parameters in a gene expression experiment. There were many aspects to consider in the model for the analysis of the constitutive difference that did not detect more fine-tuned strain differences in the oldest age group, highlighted in the second experiment. These results may show that the higher expression of some of the ABC transporters tested is part of the resistance profile of PR mosquitoes, but it would have to be observed in males as well since they are also pyrethroid-resistant, and at all age groups^42^, which was not the case in the previous experiment.

This follow-up transcript expression assay also revealed that the exposure to deltamethrin did not have a significant effect on the expression of the genes of interest. This could be the result of using the LD_25_, respective to each strain, which may be too low at the 2 h timepoint to elicit a strong stress response and significantly alter transcript expression. De Marco *et al*.^37^ studied the expression dynamics of detoxification genes and ABC transporter genes following exposure to permethrin in *An. stephensi*. It was demonstrated that the response involved upregulation of certain genes while others were downregulated, and that this pattern would change over time between 6 and 48 h. Consequently, time is an important factor to account for with gene expression experiments and the timepoint we investigated may not have been the most representative of the expression changes related to ABC transporter gene expression. A time course experiment may provide more information about our genes of interest in a future study. Alternatively, our results could also indicate that the genes of interest are not involved in the response to deltamethrin exposure, which does not eliminate a role of ABC transporters in defense against pyrethroids, following the results of the toxicity assays.

The exposure to deltamethrin in combination with ABC transporter modulators highlighted differences between the susceptible and resistant strains, which could imply a different dynamic and function of ABC transporters in the two strains. The effect of the two main drugs used in the toxicity assays can give an idea of the differences between strains, but also a better understanding of the function of dexamethasone compared to verapamil. Verapamil is a competitive inhibitor of ABC transporter substrates, since it is itself a substrate of ABC transporters^49^, as well as a calcium channel blocker^43^. It stimulates ATPase activity while preventing efflux at the transporter level, which increases insecticide toxicity^56^. Verapamil was extensively used to determine the involvement of ABC transporters in the export of drugs and resistance to chemotherapy for instance, and to other drugs in mammals^57–60^ and in insects^34,35,39–41,56,61,62^. Verapamil had a synergistic effect on deltamethrin-induced mortality in Rock mosquitoes, regardless of pretreatment time, but it did not significantly affect mortality in PR mosquitoes. The increasing mortality trend after 60 minutes of exposure to deltamethrin may however indicate that verapamil is slow to act in PR mosquitoes.

Dexamethasone has been described in mammals as a stimulant of ABC transporter expression, as well as a substrate of ABCB and ABCG transporters, especially those involved in cancer resistance^44,45^, and is readily transported by MDR1 in mouse brain and human cells^46,48^. Thus, verapamil and dexamethasone share the ability to be transported by ABC transporters, hence, potentially sharing a similar role. In arthropods, however, it was thought to be an inducer of cytochrome P450 expression^63^. In *Pediculus humanus* for instance, dexamethasone induced tolerance to ivermectin, whereas verapamil increased ivermectin toxicity^63^, which led the authors to hypothesize that decreasing ABC transporter export with verapamil would increase toxicity, and that dexamethasone ultimately increased P450 activity. However, based on the results obtained in the present study using PR and Rock mosquitoes, as well as previous work^42^, increased P450 activity induces tolerance to deltamethrin and is likely part of the resistance mechanism in PR mosquitoes, whereas the toxicity assay exposing PR mosquitoes to deltamethrin did not increase survival in presence of dexamethasone. Survival was decreased when mosquitoes were exposed to these drugs, suggesting that increased P450 is either not the major modification induced by the exposure to dexamethasone, or is not the effect of this drug in *Ae. aegypti*. The effect of dexamethasone suggests that in *Ae. aegypti*, its role is more likely similar to the role of verapamil in Rock mosquitoes and can act as an ABC transporter competitive inhibitor. Dexamethasone and verapamil may differ in certain functional aspects, which would explain the divergence of results in PR after a 4 h pretreatment, as well as the temporal dimension of the effect of dexamethasone. Additionally, the results of the CNS preparation with exposure to dexamethasone show that it decreased the IC_50_ of deltamethrin by increasing firing rate at the nerve, which suggests a direct role of dexamethasone at the blood brain barrier. Dexamethasone also significantly decreased the time needed for deltamethrin to induce nerve death.

The results highlight that ABC transporters may not play an important role in the efflux of deltamethrin in the PR strain, and their involvement would be delayed compared to Rock. It can be hypothesized that since PR mosquitoes carry mutations that confer target-site insensitivity^22^ and exhibit increased P450 activity^42^, this strain may not rely as heavily on ABC transporter export compared to the susceptible Rock strain. The resistance mechanism would be relying on P450 activity instead^42^, which can be observed through the effect of PBO, used here as a positive control, which significantly increases mortality in both PR and Rock mosquitoes. Regarding the resistance mechanisms in PR, the expression analysis did not show a consistent higher expression of the select genes in PR or in case of exposure to deltamethrin, but the constitutive gene expression analysis did not show a decreased overall expression of the ABC transporter transcripts of interest either. The expression patterns were particularly interesting for the gene ABCB4, because it was previously found to be more highly expressed in other pyrethroid-resistant strains of mosquitoes when compared to the New Orleans susceptible strain of *Ae. aegypti*^19^, but does not follow the same pattern of expression here. Two hypotheses can be offered to explain the discrepancies between studies in terms of gene expression: (1) comparing a resistant strain to a separate susceptible strain, rather than a susceptible isogenic strain, can pose a challenge since there are likely other genetic differences between the two that could confound the results of such an experiment; (2) susceptible New Orleans and Rock strains can also present genetic differences resulting in diverging expression patterns when a resistant strain is compared. Interestingly, the expression pattern of ABCB4 may be part of a compensatory mechanism between P450s and ABC transporters, which could be selected to avoid fitness costs in resistant strains, due to a higher P450 activity that is energetically costly. However, if such a mechanism exists, then more experiments would need to be conducted to gain an understanding of this phenomenon and verify our hypothesis.

Overall, the competitive inhibition of ABC transporter export function by verapamil, and potentially dexamethasone, increased mortality, although no expression level change after exposure to deltamethrin was detected. These can be interpreted as discrepancies between mosquito species or insecticide classes, since the genes chosen for this study were either involved in response to other modes of action for *Aedes* species^39,40^, or extracted from work done with *An. stephensi* exposed to permethrin^35,36,38^, and may not be involved in the response to deltamethrin exposure. ABC transporter genes involved in the response to deltamethrin may be different from those genes examined in this study and their differential expression could explain the survival curves observed for Rock, and PR to a lesser extent. Consequently, this study contributes to the large body of work demonstrating the heterogeneity of the role of ABC transporters in insecticidal challenge^64^, especially across species. Overall, we observed differences between strains due to treatment with ABC transporter modulators, illustrated by the effect of verapamil in particular. There is also a temporal aspect to their contribution towards deltamethrin challenge, especially in PR, and in the expression of the tested ABC transporters, which was variable between age groups. Additionally, a different role in females and males was suggested by the differences between sexes, as previously observed^36,38^.

Although the genes involved in deltamethrin export were not clearly delineated, the toxicity assays in *Ae. aegypti*, complemented by the CNS preparation study in *D. melanogaster*, highlighted the participation of ABC transporters in deltamethrin cellular export, with an increased efficacy when using competitive inhibitors in Rock, and potentially in PR. This work also provided insight into the function of dexamethasone when applied in conjunction with deltamethrin, presumably allowing increased penetration, and eventually, increased nerve inhibition and death. These findings highlight the complicated nature of insecticide resistance by demonstrating the likely role of other proteins involved in insecticide detoxification or transport, emphasizing the need to continue investigating the mechanisms that confer resistance in different species.

## Methods

### Subjects

#### *Aedes aegypti*

Two strains of mosquitoes were used in this study: (1) a susceptible laboratory population, Rockefeller (Rock), established in 1937 (obtained through BEI Resources, NIAID, NIH: *Aedes aegypti*, Strain ROCK, MRA-734, contributed by David W. Severson), and (2) a pyrethroid-resistant strain, Puerto Rico (PR), collected from San Juan, Puerto Rico and maintained in the laboratory under selection pressure since 2012 (provided by Centers for Disease Control and Prevention (CDC) for distribution by BEI Resources, NIAID, NIH: *Aedes aegypti*, Strain Puerto Rico, Eggs, NR-48830). Our mosquito-rearing protocol can be found in Rault *et al*.^42^. Mosquitoes were aspirated and transferred to a new cage within 24 h of emergence in order to maintain age-matched cohorts of each strain. Mosquitoes were anesthetized on ice and females and males were separated for experiments. Females and males of three age groups, 1 to 3 d old, 3 to 5 d old, and 5 to 7 d old, were collected for RNA extraction and subsequent gene expression analysis, whereas female mosquitoes of the two strains were collected 5 to 7 d after emergence for toxicity assays and gene expression confirmation.

#### *Drosophila melanogaster*

The wildtype Oregon-R (OR) strain of *Drosophila melanogaster* was used for CNS recordings. The OR strain was provided by Dr. Jeffrey Bloomquist at the University of Florida and was originally donated by Doug Knipple, Cornell University, Ithaca NY, USA. All fly strains were maintained in culture at Louisiana State University Agricultural Center (Department of Entomology) and were reared on standard medium in *Drosophila* tubes at 25 °C, 12:12 (light:dark) photoperiod and 55% relative humidity. For dissection, flies were anaesthetized by chilling on ice and decapitated before dissecting out CNS in Schneider’s medium (Invitrogen, Paisley, Scotland, UK).

### Gene expression

#### Deltamethrin exposure

Two experiments were performed in order to 1) identify constitutive differences between susceptible and resistant strains, as well as sex and age differences, in the expression of ABC transporter transcripts, and 2) identify deltamethrin-induced changes in the expression of the same transcripts. The specimens used were either 1) untreated, in triplicates of 10 mosquitoes of the 2 strains, of 3 age groups for both sexes, or 2) 5 to 7 d old females that received their respective LD_25_ (at 24 h) calculated based on weight differences observed by strain (resistant or susceptible). Deltamethrin was applied topically on the pronotum, then mosquitoes were placed in Petri dishes in groups of 10. Each condition was repeated in 3 replicates. Rock females received 0.2 µL of a stock solution of 22.5 nM in acetone and PR females received 0.2 µL of a stock solution of 1.5 µM of deltamethrin in acetone. The specimens were placed on ice before recovery after a 2 h exposure, and subsequently flash-frozen in liquid nitrogen and stored at −80 °C.

#### RNA extraction and cDNA synthesis

The RNA extraction area and materials were treated with RNase AWAY™ (Molecular BioProducts™, San Diego, CA). RNA extraction was performed using Tri Reagent RT (Molecular Research Center, Inc., Cincinnati, OH) and 1 µg of RNA per 20 µL reaction volume was reverse transcribed using the iScript cDNA reverse transcription kit following manufacturer indications (BioRad, Hercules, CA). The resulting cDNA was diluted 2-fold (constitutive experiment) or 3-fold (deltamethrin exposure) to accommodate the volumes of reaction.

#### Quantitative reverse transcriptase polymerase chain reaction

The primers for ABCB2, ABCB4, ABCB6, and ABCG4 genes were originally designed for *An. stephensi*^35,36,38^, thus, the sequences were matched in the Basic Local Alignment Search Tool “BLAST”^65^ to find the homologous sequence in *Ae. aegypti* and design custom primers with Primer 3^66^. Additionally, Porretta *et al*.^39^ produced degenerate primers for *Ae. caspius* that also work in *Ae. aegypti* due to the conserved Walker A- Walker B region across species. Other primer sequences were gathered from Bariami *et al*.^19^ (Supplementary Table 3). All primers were synthesized by Sigma-Aldrich (Saint Louis, MO).

The quantitative reverse transcriptase (qRT) polymerase chain reaction (PCR) experiments were performed using a BioRad CFX Connect Real-Time System with iTaq Universal SYBR® Green Supermix (BioRad). The PCR cycle protocol program consisted of an initial step of 3 min at 95 °C for denaturation, followed by 40 cycles of the following steps: 10 s at 95 °C for denaturation, 30 s at 59 °C for primer annealing, and 30 s at 72 °C for primer extension. After a final step of 72 °C for 5 min, a melting curve was taken from 65 °C to 95 °C by increments of 0.5 °C for 5 s. All reactions consisted of 2 µL of cDNA, 0.5 µL of each primer at 10 µM, 5 µL of SYBR® green and 2 µL of water, for a total volume of 10 µL. Each condition was repeated in technical triplicates.

The statistical analysis of the qRT-PCR results was performed using the R^67^ package MCMC.qpcr^68^. This method uses a generalized linear mixed model (GLMM). GLMM permits the analysis of complex designs by an ANOVA-type analysis. The model also considers primer efficiencies and allows the calculation of molecule counts from C*t* values for all conditions. Prior to analysis, outliers of technical replicates were removed after visualization of the melt curve and amplification curve. All transcripts of interest and two housekeeping genes were analyzed. The housekeeping genes selected for the analysis were β-actin and ribosomal protein S17 (RPS17)^69^, however, these genes were not specified as “control” genes since no assumption was made about their stability, which is one of the features of the package used. The effect of (1) age, sex, and strain, or (2) strain and treatment, on the expression of the selected genes was measured. *P*-values were corrected for multiple tests by applying the method of Benjamini and Hochberg^70^ with a false discovery rate of 5%.

### Toxicity bioassays

The reagents used were acetone (Sigma Aldrich, Saint Louis, MO), verapamil hydrochloride, 99% (ThermoFisher Scientific, NJ), dexamethasone (Sigma Aldrich, Saint Louis, MO), and technical grade deltamethrin (Chem Service, Inc., PA).

A toxicity bioassay was performed with two pretreatment times: (1) Rock and PR female mosquitoes between 5 and 7 d old received a topical application of 0.2 µL of either acetone as a control treatment, PBO as a control (10 mM), verapamil (10 mM), or dexamethasone (8 mM), diluted in acetone, on the pronotum, and were immediately placed in bottles coated with deltamethrin. A sub-lethal concentration of PBO, verapamil and dexamethasone was used for each bioassay. Rock were exposed to 0.1 mg/bottle (1 mL/bottle) and PR were exposed to 1 mg/bottle (1 mL/bottle). (2) All conditions were identical, but the topical application of the four compounds tested was performed 4 h before the transfer into the coated bottles. Controls were running simultaneously to account for natural mortality. Six replications were used for all the conditions. Mortality was recorded every 15 min for 2 h. The resulting survival curves were compared between treatments, separately for each pretreatment time and strain, and analyzed with a log-rank Mantel-Cox test. All statistical tests were carried out at a significance level of *P* ≤ 0.05. Analyses were performed with GraphPad Prism version 8.00 for Windows (GraphPad Software, La Jolla California USA, www.graphpad.com).

### Electrophysiological studies of *Drosophila melanogaster* neural systems

Neurophysiological recordings were performed on the CNS of 3^rd^-instar *D. melanogaster* larvae and slightly modified from previously described methods^50,51,71,72^. One primary difference in this study is the recordings were performed on intact CNS to ensure the blood brain barrier was fully functional. The CNS was excised from the larvae and placed in a separate dish with physiological saline (200 µL) containing: 157 mM NaCl, 3 mM KCl, 2 mM CaCl_2_, and 4 mM HEPES, pH = 7.25. Peripheral nerve trunks were drawn into a recording suction electrode and electrical activity was monitored from descending nerves originating from the ventral ganglia, with amplification by an AC/DC amplifier (Model 1700, A-M Systems, Inc., Carlsborg, WA, USA). Descending electrical activity was subjected to window amplitude discrimination and converted on-line into a rate plot, expressed in Hertz (Hz), using LabChart7 Pro (ADInstruments, Colorado Springs, CO, USA). Noise (60 Hz) was eliminated using Hum Bug (A-M Systems, Sequim, WA, USA). Activity was monitored for a 5 min time period to establish a constant baseline firing rate, as the spike frequency typically increased from 0 to 10 min before stabilization. After a baseline was established, the CNS preparation was directly exposed to test compounds by adding 200 µL of solution to the bath containing 200 µL of saline. The final concentration of solvent in the bath was 0.1% DMSO. Frequencies were measured for 3–5 min for each concentration prior to the addition of the next drug concentration. Mean spike frequencies over the 3–5 min recording period for each concentration were used to construct concentration-response curves in order to determine IC_50_ values, which were calculated by nonlinear regression (variable slope) using GraphPad Prism^TM^ (GraphPad Software, San Diego, CA, USA). Each drug concentration was replicated 5–10 times.

## Supplementary information

Supplementary tables

## Data Availability

The datasets generated during and/or analyzed during the current study are contained within the paper and its Supplementary Information Files, or can be obtained from the corresponding author on reasonable request.

## References

[1] Kraemer MU (2015). The global distribution of the arbovirus vectors *Aedes aegypti* and *Ae. albopictus*. eLife.

[2] Centers for Disease Control and Prevention. Zika Virus. *CDC* https://www.cdc.gov/zika/vector/range.html (2017).

[3] Kyle JL, Harris E (2008). Global Spread and Persistence of Dengue. Annu. Rev. Microbiol..

[4] Muktar, Y., Tamerat, N. & Shewafera, A. *Aedes aegypti* as a Vector of Flavivirus. *J. Trop. Dis. Public Health* **4** (2016).

[5] Powers AM, Logue CH (2007). Changing patterns of chikungunya virus: re-emergence of a zoonotic arbovirus. J. Gen. Virol..

[6] Coffey LL, Failloux A-B, Weaver SC (2014). Chikungunya Virus–Vector Interactions. Viruses.

[7] Duffy MR (2009). Zika Virus Outbreak on Yap Island, Federated States of Micronesia. N. Engl. J. Med..

[8] Musso D, Nilles EJ, Cao-Lormeau V-M (2014). Rapid spread of emerging Zika virus in the Pacific area. Clin. Microbiol. Infect..

[9] Tomori O (2004). Yellow Fever: The Recurring Plague. Crit. Rev. Clin. Lab. Sci..

[10] Ranson, H., Burhani, J., Lumjuan, N. & Black, W. C. I. Insecticide resistance in dengue vectors. *Trop. Online* **1** (2010).

[11] Vontas J (2012). Insecticide resistance in the major dengue vectors *Aedes albopictus* and *Aedes aegypti*. Pestic. Biochem. Physiol..

[12] Smith LB, Kasai S, Scott JG (2016). Pyrethroid resistance in *Aedes aegypti* and *Aedes albopictus*: Important mosquito vectors of human diseases. Pestic. Biochem. Physiol..

[13] Zaim M, Aitio A, Nakashima N (2000). Safety of pyrethroid-treated mosquito nets. Med. Vet. Entomol..

[14] Hougard J-M (2003). Comparative performances, under laboratory conditions, of seven pyrethroid insecticides used for impregnation of mosquito nets. Bull. World Health Organ..

[15] Manjarres-Suarez A, Olivero-Verbel J (2013). Chemical control of *Aedes aegypti*: a historical perspective. Rev. Costarric. Salud Pública.

[16] Brengues C (2003). Pyrethroid and DDT cross-resistance in *Aedes aegypti* is correlated with novel mutations in the voltage-gated sodium channel gene. Med. Vet. Entomol..

[17] Du, Y., Nomura, Y., Zhorov, B. S. & Dong, K. Sodium Channel Mutations and Pyrethroid Resistance in *Aedes aegypti*. *Insects* **7** (2016). 10.3390/insects7040060 PMC5198208 27809228

[18] Strode C (2008). Genomic analysis of detoxification genes in the mosquito *Aedes aegypti*. Insect Biochem. Mol. Biol..

[19] Bariami V, Jones CM, Poupardin R, Vontas J, Ranson H (2012). Gene Amplification, ABC Transporters and Cytochrome P450s: Unraveling the Molecular Basis of Pyrethroid Resistance in the Dengue Vector, *Aedes aegypti*. PLoS Negl. Trop. Dis..

[20] Reid WR (2014). Transcriptional Analysis of Four Family 4 P450s in a Puerto Rico Strain of *Aedes aegypti* (Diptera: Culicidae) Compared With an Orlando Strain and Their Possible Functional Roles in Permethrin Resistance. J. Med. Entomol..

[21] Dusfour I (2015). Deltamethrin Resistance Mechanisms in *Aedes aegypti* Populations from Three French Overseas Territories Worldwide. PLoS Negl. Trop. Dis..

[22] Estep AS, Sanscrainte ND, Waits CM, Louton JE, Becnel JJ (2017). Resistance Status and Resistance Mechanisms in a Strain of *Aedes aegypti* (Diptera: Culicidae) From Puerto Rico. J. Med. Entomol..

[23] Buss DS, Callaghan A (2008). Interaction of pesticides with p-glycoprotein and other ABC proteins: A survey of the possible importance to insecticide, herbicide and fungicide resistance. Pestic. Biochem. Physiol..

[24] Dermauw W, Van Leeuwen T (2014). The ABC gene family in arthropods: Comparative genomics and role in insecticide transport and resistance. Insect Biochem. Mol. Biol..

[25] Higgins CF (1992). ABC transporters: From Microorganisms to Man. Annu. Rev. Cell Biol..

[26] Heckel DG (2012). Learning the ABCs of *Bt*: ABC transporters and insect resistance to *Bacillus thuringiensis* provide clues to a crucial step in toxin mode of action. Pestic. Biochem. Physiol..

[27] Juliano RL, Ling V (1976). A surface glycoprotein modulating drug permeability in Chinese hamster ovary cell mutants. Biochim. Biophys. Acta BBA - Biomembr..

[28] Ueda K, Cardarelli C, Gottesman MM, Pastan I (1987). Expression of a full-length cDNA for the human ‘MDR1’ gene confers resistance to colchicine, doxorubicin, and vinblastine. Proc. Natl. Acad. Sci..

[29] Castanys-Muñoz E, Alder-Baerens N, Pomorski T, Gamarro F, Castanys S (2007). A novel ATP-binding cassette transporter from *Leishmania* is involved in transport of phosphatidylcholine analogues and resistance to alkyl-phospholipids. Mol. Microbiol..

[30] Castanys-Muñoz E, Pérez-Victoria JM, Gamarro F, Castanys S (2008). Characterization of an *ABCG*-like transporter from the protozoan parasite *Leishmania* with a role in drug resistance and transbilayer lipid movement. Antimicrob. Agents Chemother..

[31] Epel D (2008). Efflux Transporters: Newly Appreciated Roles in Protection against Pollutants. Environ. Sci. Technol..

[32] Kusuhara H, Sugiyama Y (2006). ATP-binding cassette, subfamily G (ABCG family). Pflüg. Arch. - Eur. J. Physiol..

[33] Tarr PT, Tarling EJ, Bojanic DD, Edwards PA, Baldán Á (2009). Emerging new paradigms for *ABCG* transporters. Biochim. Biophys. Acta BBA - Mol. Cell Biol. Lipids.

[34] Buss DS, Mccaffery AR, Callaghan A (2002). Evidence for P-glycoprotein modification of insecticide toxicity in mosquitoes of the *Culex pipiens* complex. Med. Vet. Entomol..

[35] Epis Sara, Porretta Daniele, Mastrantonio Valentina, Comandatore Francesco, Sassera Davide, Rossi Paolo, Cafarchia Claudia, Otranto Domenico, Favia Guido, Genchi Claudio, Bandi Claudio, Urbanelli Sandra (2014). ABC transporters are involved in defense against permethrin insecticide in the malaria vector Anopheles stephensi. Parasites & Vectors.

[36] Epis S (2014). Temporal dynamics of the ABC transporter response to insecticide treatment: insights from the malaria vector *Anopheles stephensi*. Sci. Rep..

[37] De Marco L (2017). The choreography of the chemical defensome response to insecticide stress: insights into the *Anopheles stephensi* transcriptome using RNA-Seq. Sci. Rep..

[38] Mastrantonio V (2017). Gene expression modulation of ABC transporter genes in response to permethrin in adults of the mosquito malaria vector *Anopheles stephensi*. Acta Trop..

[39] Porretta D (2008). Defence mechanisms against insecticides temephos and diflubenzuron in the mosquito *Aedes caspius*: the P-glycoprotein efflux pumps. Med. Vet. Entomol..

[40] Figueira-Mansur J (2013). Silencing of P-glycoprotein increases mortality in temephos-treated *Aedes aegypti* larvae. Insect Mol. Biol..

[41] Lima EP (2014). Evaluation of the role of ATP-binding cassette transporters as a defence mechanism against temephos in populations of *Aedes aegypti*. Mem. Inst. Oswaldo Cruz.

[42] Rault LC, O’Neal ST, Johnson EJ, Anderson TD (2019). Association of age, sex, and pyrethroid resistance status on survival and cytochrome P450 gene expression in *Aedes aegypti* (L.). Pestic. Biochem. Physiol..

[43] Bergson P, Lipkind G, Lee SP, Duban M-E, Hanck DA (2011). Verapamil Block of T-Type Calcium Channels. Mol. Pharmacol..

[44] Manceau S (2012). Expression and induction by dexamethasone of ABC transporters and nuclear receptors in a human T-lymphocyte cell line. J. Chemother..

[45] Marquez, B. & Van Bambeke, F. *ABC Multidrug Transporters: Target for Modulation of Drug Pharmacokinetics and Drug-Drug Interactions*. vol. 12 (Bentham Science Publishers Ltd., 2011). 10.2174/138945011795378504 21039335

[46] Schinkel AH, Wagenaar E, van Deemter L, Mol CA, Borst P (1995). Absence of the mdr1a P-Glycoprotein in mice affects tissue distribution and pharmacokinetics of dexamethasone, digoxin, and cyclosporin A. J. Clin. Invest..

[47] Narang VS (2008). Dexamethasone increases expression and activity of multidrug resistance transporters at the rat blood-brain barrier. Am. J. Physiol. - Cell Physiol..

[48] Ueda K, Taguchi Y, Morishima M (1997). How does P-glycoprotein recognize its substrates?. Semin. Cancer Biol..

[49] Kim RB (2002). Drugs as P-glycoprotein substrates, inhibitors, and inducers. Drug Metab. Rev..

[50] Chen R (2019). Functional Coupling of K+–Cl– Cotransporter (KCC) to GABA-Gated Cl– Channels in the Central Nervous System of *Drosophila melanogaster* Leads to Altered Drug Sensitivities. ACS Chem. Neurosci..

[51] Swale DR, Sun B, Tong F, Bloomquist JR (2014). Neurotoxicity and Mode of Action of N, N-Diethyl-Meta-Toluamide (DEET). PLOS ONE.

[52] Francis SAM, Taylor-Wells J, Gross AD, Bloomquist JR (2017). Toxicity and Physiological Actions of Carbonic Anhydrase Inhibitors to *Aedes aegypti* and *Drosophila melanogaster*. Insects.

[53] Merzendorfer, H. Chapter One - ABC Transporters and Their Role in Protecting Insects from Pesticides and Their Metabolites. In *Advances in Insect Physiology* (ed. Cohen, E.) vol. 46 1–72 (Academic Press, 2014).

[54] Koehn LM (2019). Developmental differences in the expression of ABC transporters at rat brain barrier interfaces following chronic exposure to diallyl sulfide. Sci. Rep..

[55] Suzuki T (2006). Gender-related differences in expression and function of hepatic P-glycoprotein and multidrug resistance-associated protein (Mrp2) in rats. Life Sci..

[56] Podsiadlowski L, Matha V, Vilcinskas A (1998). Detection of a P-glycoprotein related pump in *Chironomus* larvae and its inhibition by verapamil and cyclosporin A. Comp. Biochem. Physiol. B Biochem. Mol. Biol..

[57] Chatterjee M, Robson CN, Harris AL (1990). Reversal of Multidrug Resistance by Verapamil and Modulation by α1-Acid Glycoprotein in Wild-type and Multidrug-resistant Chinese Hamster Ovary Cell Lines. Cancer Res..

[58] Tsuruo T, Iida H, Tsukagoshi S, Sakurai Y (1981). Overcoming of Vincristine Resistance in P388 Leukemia *in Vivo* and *in Vitro* through Enhanced Cytotoxicity of Vincristine and Vinblastine by Verapamil. Cancer Res..

[59] Xu J (2018). Verapamil Increases the Bioavailability and Efficacy of Bedaquiline but Not Clofazimine in a Murine Model of Tuberculosis. Antimicrob. Agents Chemother..

[60] Belpomme D (2000). Verapamil increases the survival of patients with anthracycline-resistant metastatic breast carcinoma. Ann. Oncol..

[61] Chouaïbou M, Zivanovic GB, Knox TB, Jamet HP, Bonfoh B (2014). Synergist bioassays: A simple method for initial metabolic resistance investigation of field *Anopheles gambiae* s.l. populations. Acta Trop..

[62] Strycharz JP (2013). Resistance in the highly DDT-resistant 91-R strain of *Drosophila melanogaster* involves decreased penetration, increased metabolism, and direct excretion. Pestic. Biochem. Physiol..

[63] Yoon KS (2011). Brief exposures of human body lice to sub-lethal amounts of ivermectin over transcribes detoxification genes involved in tolerance. Insect Mol. Biol..

[64] Porretta D (2016). How heterogeneous is the involvement of ABC transporters against insecticides?. Acta Trop..

[65] Altschul SF, Gish W, Miller W, Myers EW, Lipman DJ (1990). Basic local alignment search tool. J. Mol. Biol..

[66] Rozen, S. & Skaletsky, H. Primer3 on the WWW for general users and for biologist programmers. In *Bioinformatics Methods and Protocols* (eds. Misener, S. & Krawetz, Stephen A.) 365–386, 10.1385/1-59259-192-2:365 (Humana Press, 2000). 10.1385/1-59259-192-2:365 10547847

[67] R Core Team. *R: A language and environment for statistical computing*. (R Foundation for Statistical Computing, Vienna, Austria, 2013).

[68] Matz MV, Wright RM, Scott JG (2013). No control genes required: Bayesian analysis of qRT-PCR data. PLoS ONE.

[69] Dzaki N, Ramli KN, Azlan A, Ishak IH, Azzam G (2017). Evaluation of reference genes at different developmental stages for quantitative real-time PCR in *Aedes aegypti*. Sci. Rep..

[70] Benjamini Y, Hochberg Y (1995). Controlling the false discovery rate: A practical and powerful approach to multiple testing. J. R. Stat. Soc. Ser. B Methodol..

[71] Chen R, Swale DR (2018). Inwardly Rectifying Potassium (Kir) Channels Represent a Critical Ion Conductance Pathway in the Nervous Systems of Insects. Sci. Rep..

[72] Swale, D. R., Gross, A. D., Coquerel, Q. R. R. & Bloomquist, J. R. Electrophysiological Recording of The Central Nervous System Activity of Third-Instar *Drosophila Melanogaster*. *J. Vis. Exp. JoVE*, 10.3791/58375 (2018). 10.3791/58375 30531714

