# Supplementary material for: Age- and sex-related ABC transporter expression in pyrethroid-susceptible and –resistant Aedes aegypti
Source: Sci Rep. 2019 Dec 20;9:19551. doi: 10.1038/s41598-019-56134-2 (PMC6925122; doi:10.1038/s41598-019-56134-2)
Supplement: Supplementary file 1 — Supplementary tables [file 41598_2019_56134_MOESM1_ESM.doc]

**Supplementary material**

**Age- and sex-related ABC transporter expression in pyrethroid-susceptible and -resistant**

***Aedes aegypti***

Authors: Leslie C. Rault^1*^, Ellis J. Johnson^1^, Scott T. O’Neal^1^, Rui Chen^2^, Sarah E. McComic^2^, Daniel R. Swale^2^, Troy D. Anderson^1^

^1^ Department of Entomology, University of Nebraska, 103 Entomology Hall, Lincoln, NE 68583

^2^ Department of Entomology, Louisiana State University AgCenter, Baton Rouge, LA 70803

*corresponding author: lrault2@unl.edu

**Supplementary Table 1: Significant factors in the expression of the genes tested in the quantitative reverse transcriptase polymerase chain reaction experiment comparing age, sex, and strain. The baseline factor levels were age 1 to 3 d old for the age factor, female for the sex factor, and Rockefeller for the strain factor. “Age (5 to 7 d old)” refers to the difference between 1 to 3 d old and 5 to 7 d old. Significance level codes: “***” Adjusted P-value <0.001; “**” adjusted P-value between 0.001 and 0.01; “*” adjusted P-value between 0.01 and 0.05.**

| **Gene** | **Factor** | **Fold change** | **Adjusted *P*-value and significance level** | |
| --- | --- | --- | --- | --- |
|  |  | **Genes of interest** |  |  |
| ABCG4 | age (5 to 7 d old) | 71.4-fold decrease in 5-7 DO | 8.88E-15 | *** |
| ABCB4 | Interaction strain by sex | 3.4-fold decrease in males between strains | 0.037277 | * |
| ABCB6 | age (5 to 7 d old) | 7.1-fold decrease in 5-7 DO | 1.15E-05 | *** |
|  | sex | 5.6-fold decrease in males | 0.000269 | *** |
| dABCB | Interaction age (5 to 7 d old) by sex | 10.5-fold decrease in males 5-7 DO | 0.000349 | *** |
|  | sex | 8.5-fold decrease in males | 1.02E-06 | *** |
| Pgp | sex | 2.5696 | 0.013193 | * |
|  |  | **Housekeeping genes** |  |  |
| Actin | age (5 to 7 d old) | 0.2561 | 0.000168526 | *** |
|  | Interaction age (5 to 7 d old) by sex | 0.1824 | 0.000630803 | *** |
| RPS17 | sex | 0.4635 | 0.045097079 | * |
|  | Interaction age (5 to 7 d old) by sex | 0.2305 | 0.002678649 | ** |

Supplementary Table 2: Significant factors in the expression of the genes tested in the quantitative reverse transcriptase polymerase chain reaction experiment comparing strains and treatment (acetone versus deltamethrin). The baseline factor levels were Rockefeller (Rock) for the strain factor and control (acetone) for the treatment factor. Significance level codes: “***” Adjusted P-value <0.001; “**” adjusted P-value between 0.001 and 0.01; “*” adjusted P-value between 0.01 and 0.05.

| **Gene** | **Factor** | **Fold change** | **Adjusted *P*-value** | **Significance level** |
| --- | --- | --- | --- | --- |
| **Genes of interest** | | | | |
| dABCB | Strain | 5.751995 | 0.000852 | *** |
| ABCB2 | Strain | 2.898752 | 0.010562 | * |
| ABCG4 | Strain | 3.40373 | 0.010562 | * |
| ABCB4 | Strain | 2.6-fold decrease in Rock | 0.024645 | * |
| ABCB6 | Strain | 2.868046 | 0.024984 | * |
| **Housekeeping genes** | | | | |
| RPS17 | Strain | 2.106099 | 0.033993 | * |
| Actin | Strain | 30.31744 | 0 | *** |
| Actin | Treatment | 2.557122 | 0.024645 | * |

Supplementary Table 3: List of primers, the genes they are associated with, product size, efficiency, and origin.

| **Gene** | **Forward primer** | **Reverse primer** | **Product length** | **Efficiency** | **Origin** |
| --- | --- | --- | --- | --- | --- |
| **Actin** | CGTTCGTGACATCAAGGAAA | GAACGATGGCTGGAAGAGAG | 175 bp | 98.8% | 69 |
| **RPS17** | AAGAAGTGGCCATCATTCCA | GGTCTCCGGGTCGACTTC | 200 bp | 95% | 69 |
| **ABCB2** | ATATCCGAACCGTTGCAAGC | TTGGAATTGTTGGTCGCGTT | 450 bp | 93.3% | Custom primers |
| **ABCB4** | GAATGGCCGCATCTGCCAG | CGTTTCCTTGGGACCGAGCT | 167 bp | 105.8% | 19 |
| **ABCB6** | TACCTGTTTTCCCATCGGCT | CATTGCTCGGACAGTACTGC | 108 bp | 112% | Custom primers |
| **P-glycoprotein “Pgp”** | TGGAAAAGTTGACATTGACGG | TGCGATCAAACAGAACAGGTT | 104 bp | 100.8% | 40 |
| **Degenerate ABCB “dABCB”** | AAGGKSARACGSTBGCCCTGGTTGGA | GAGGTBGCYTCGTCCAGCAGVAGGA | 500 bp gDNA | 115% | 39 |
| **ABCG4** | TCTGCACCATCCATCAACCT | AGGCCACTTCCATCACGTAA | 180 bp | 111% | Custom primers |
